# Management of Putrescent Pulp and a Permanent Root Filling

**Published:** 1908-08-15

**Authors:** T. C. Reid

**Affiliations:** Adrian, Mich.


### MANAGEMENT OF PUTRESCENT PULP AND A PERMANENT ROOT FILLING.

BY DR. T. C. REID, ADRIAN, MICH.

Read before the Southwestern Michigan and Fifth District Dental Societies, Grand Rapids, Mich., April 14 and 15, 1908.

In presenting this paper I do not lay claim to anything particularly new, to the members of this society. Rather laying before you for approval or otherwise, as the case may be, some thoughts that may be of use to us in our daily practice.

In no other branch of our profession are the conditions so varied or the treatments so diversified as in connection with pulpless teeth.

In commencing the treatment of such teeth, the first query in each case which presents itself is as to the manner in which the vitality has been lost. We consider pulpless teeth under two classes.

First, those which have died through mechanical causes and thermal changes.

Second, those dying from infection through decay, subjecting the canal contents to inflammation, suppuration and death.

In the treatment of teeth under class one I apply the rubber dam before an opening is secured into the canal so that all danger of further infection is obviated. I then wash out the canal with warm water and dry thoroughly with alcohol, being careful not to disturb the contents of the canal any more than possible; then place in cavity a small piece of cotton saturated with equal parts of beechwood creosote and formalin. After three or four days reopen canal, with rubber applied, wash out with warm water and with a fine broach remove the contents of the canal thoroughly and repeat the treatment with creosote and formalin for some length of time as in the first, then cleanse the canal with a 40 per cent solution of sulphuric acid when they may be safely and permanently sealed.

Class two. Those dying from infection through decay, subjecting the canal contents to inflammation, suppuration and death. If under this head there should be a blind abscess with tooth to be treated, I first wash out well with a warm solution of glyco-thymoline, open up as thoroughly as possible, apply rubber dam and dry well with alcohol. Insert a loosely placed cotton dressing saturated with ¼ of 1 per cent of beechwood creosote, being careful not to seal the cavity too tightly and paint gums with aconite and resublimed iodine.

If at the end of three or four days inflammation has not subsided and pus still present, I repeat the treatment. Should there be no pus or inflammation present, I use same treatment as in case under the first class. If the abscess has a fistulous opening I wash out well with a warm solution of glyco-thymoline forcing it through the opening and dry thoroughly, then by means of a small piece of cotton on a fine broach I force ¼ of 1 per cent solution of formalin in beechwood creosote through until it appears at the opening in the gum. Dismiss patient, leaving canal cavity open, and repeat operation in three or four days. After this treatment the same method may be followed as described above, where no complication previously existed.

We are now ready for a root canal filling and desire one that is antiseptic, non-irritant and that will permanently seal the apical foramen. I have experimented with several materials, among them being chloro-percha, iodoform, and various other materials, until at last I found one which has for the past six years given me the best results. When canals are ready to be filled and all preliminary steps taken, a thread of cotton wrapped around a smooth broach, dipped into tincture of iodine, then into finely powdered tannic acid, and force to the apical end of root canal; afterward drying the canal thoroughly so that all excess of iodine is removed, wipe out with alcohol and it is ready for the filling. I use oxide of zinc mixed with $\frac{1}{4}$ of 1 per cent formalin in beechwood creosote to the consistency of a thick cream and by means of a thread of cotton on fine broach force it to end of root, using care not to force it through the apical end; repeat until the canal is well filled, then insert your gutta-percha points.

With this material sepsis will not recur, as you have a material that sets as hard as cement, sealing the apical foramen perfectly and which is antiseptic and germ proof.

